# Granulocyte Colony Stimulating Factor (G-CSF) Induced Splenic Infarction in Breast Cancer Patient Treated with Dose-Dense Chemotherapy Regimen

**DOI:** 10.1155/2019/8174986

**Published:** 2019-02-13

**Authors:** Majed A. Alshamrani, Meteb Al-Foheidi, Ahmed H. Abdulrahim

**Affiliations:** ^1^Department of Pharmaceutical Care Services, Ministry of National Guard-Health Affairs, Jeddah, Saudi Arabia; ^2^King Abdullah International Medical Research Center, Saudi Arabia; ^3^Department of Oncology, Ministry of National Guard-Health Affairs, Jeddah, Saudi Arabia

## Abstract

**Introduction:**

Granulocyte colony-stimulating factor (G-CSF) is commonly used for prevention and treatment of febrile neutropenia among solid tumor patients. It is considered an effective and relatively safe supportive care medication; however, it can cause rare and serious side effects such as spleen rupture or infarction.

**Case Presentation:**

We are reporting a case of a 27-year-old female with breast cancer who has been treated with dose-dense chemotherapy and received colony-stimulating factor as primary prevention of febrile neutropenia that was complicated halfway through with splenic infarction. This finding was confirmed by computed tomography (CT) scan and splenic biopsy. Management was conservative without the need of surgical intervention.

**Conclusion:**

Although splenic infarction is an extremely rare side effect of G-CSF, it can be a serious complication that should be recognized, monitored, and managed carefully.

## 1. Introduction

Febrile neutropenia (FN) is common with chemotherapeutic agents and considered one of the oncologic emergencies [1, 2]. It is associated with a major increase in morbidity and mortality, healthcare resource use, and compromise efficacy of chemotherapy [2–4]. Granulocyte colony-stimulating factor (G-CSF) has been shown to reduce the duration and severity of neutropenia and the risk of febrile neutropenia and enable delivery of full dose-dense chemotherapy when indicated [5–7]. G-CSF can be used as primary prophylaxis when the risk of neutropenic fever is 20% or higher with any chemotherapy. Furthermore, G-CSF can be used as secondary prophylaxis or for the treatment of established neutropenic fever [2, 7]. It is considered effective and relatively safe but it carries a risk for causing bone pain and local skin reactions at the site of injection [8]. In addition, there were few case reports of severe splenic rupture, splenic infarction, myocardial infarction, and stroke associated with use of colony-stimulating factors in both hematologic and solid tumors. For those who developed splenic complication, some of them were managed conservatively while those having other severe complications warranted splenectomy [9–12].

## 2. Case Report

**Figure 1:**
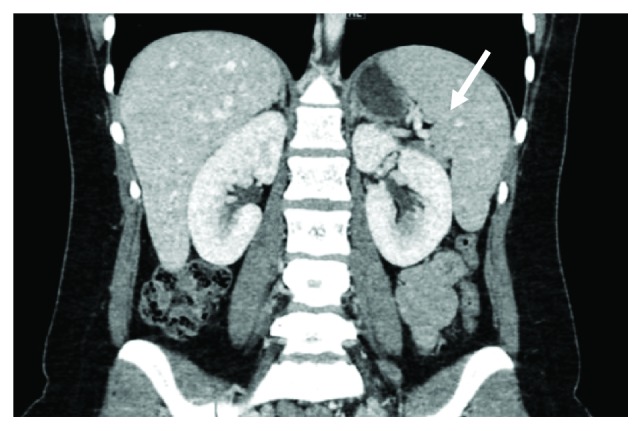
Baseline CT scan shows the normal size and appearance of the spleen before using granulocyte colony-stimulating factor (G-CSF).

**Figure 2:**
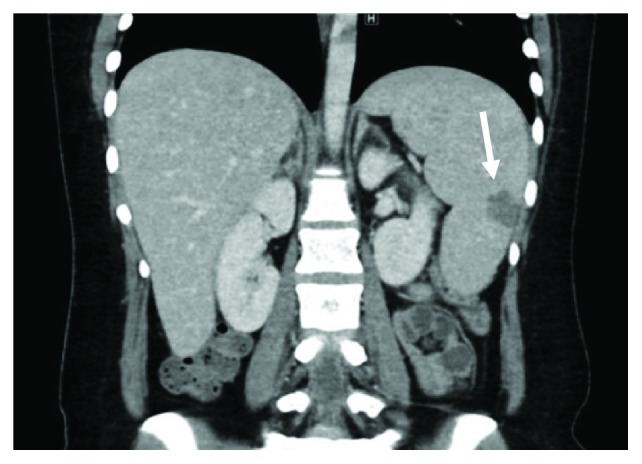
Hepatosplenomegaly with multiple splenic hypodensities consistent with splenic infarction (arrow) after using G-CSF.

**Figure 3:**
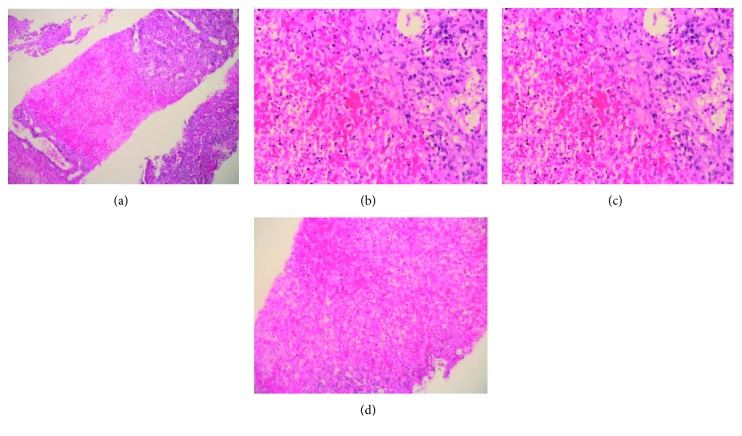
Splenic tissue with focal hemorrhagic infarction necrosis and predominantly neutrophil polymorphs. (a–d) H/E-stained section.

**Figure 4:**
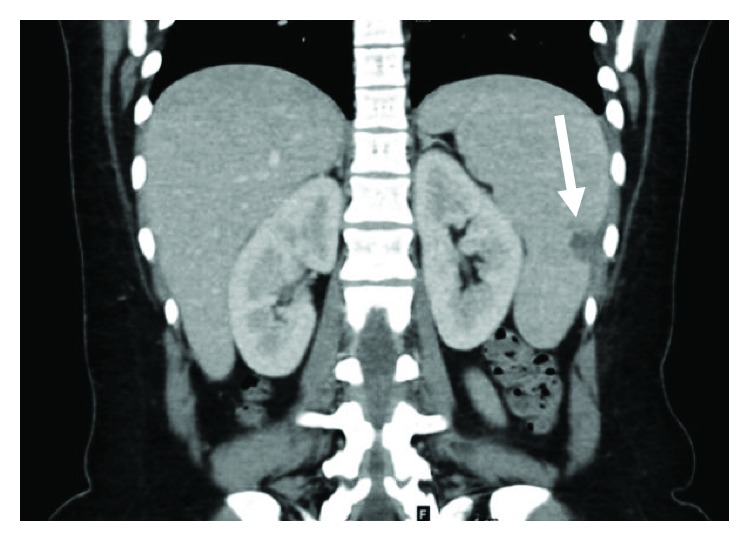
Follow-up CT scan shows hepatosplenomegaly with interval improvement regarding the splenic infarction.

A 27-year-old female patient with a known case of triple-negative breast cancer admitted to the emergency room complaining of documented fever 40°C at home which was relieved with an antipyretic. She was status post 4 cycles of neoadjuvant dose-dense AC regimen which consists of doxorubicin 60 mg/m^2^ and cyclophosphamide 600 mg/m^2^ administered every 14 days. She was prescribed primary prophylaxis filgrastim after all cycles. Physical examination was unremarkable apart from her fever. The patient was admitted to the hospital having febrile neutropenia with no focus of infection and started on empiric antibiotics and filgrastim. She had a low white blood cell (WBC) count with an absolute neutrophil count (ANC) of 1100 cells/microlitre on day 11 after cycle 4 despite being on appropriate filgrastim dose at 300 *μ*g per day for 9 days. On the second day of her admission, she recovered from neutropenia but continued to have persistent high-grade fever for almost two weeks despite escalation of the antibiotics and addition of an antifungal agent. She underwent series of investigations to identify the cause of her unexplained fever. She had extensive blood and urine cultures after each spike of fever, which all came back negative. Computed tomography (CT) scan ruled out infectious focus and showed hepatosplenomegaly with multiple splenic hypodensities and minimal perisplenic fluid which did not appear in the baseline scan (Figures 1 and 2). Infectious disease team advised for a splenic biopsy which showed splenic infarction only with no evidence of bacterial, fungal, viral, or malignant involvement (Figure 3). She also underwent an echocardiogram study, sinoscopy, and series of rheumatologic investigations that were normal. General surgery team was consulted and did not recommend any surgical intervention since the follow-up CT scan turned out negative for splenic abscesses with interval improvement in the previous splenic wedge-shaped hypodensities (Figure 4). Eventually, the patient was discharged on oral antibiotics with infectious disease and oncology clinic follow-up after being afebrile and asymptomatic for more than 72 hours.

## 3. Discussion

Granulocyte colony-stimulating factor (G-CSF) is frequently prescribed to cancer patients in order to prevent and treat febrile neutropenia secondary to the use of myelosuppressive chemotherapy. Prophylactic G-CSF use is recommended when the risk of chemotherapy-induced febrile neutropenia is >20% to maintain both dose density and dose intensity which has a major survival benefit [1, 2]. Another clinical indication of G-CSF in healthy donors of Peripheral blood stem cell transplants (PBSCT) patients or patients themselves undergoing mobilization. G-CSF use is considered effective and relatively safe but it carries a risk for causing bone pain and skin reactions at the site of injection [2]. In addition, there were a few—tough ominous—case reports of severe splenic complications including; splenic infarction or rupture in both cancer patients and healthy donors [9, 10]. Splenic complications are considered extremely rare side effects associated with the use of G-CSF, with limited number of case reported in literature [9–12]. Raut Shreeniwas et al. reported a case of splenic infarction in a 15-year-old male diagnosed with acute lymphoblastic leukemia (ALL) following treatment with filgrastim. This patient was managed conservatively with antibiotics and supportive treatment with the exclusion of filgrastim in the subsequent treatment phases [11]. Another case of spontaneous splenic rupture secondary to following prophylactic pegfilgrastim was reported by Watring et al. which was managed successfully with splenectomy [12].

To the best of our knowledge, this is a rare case where G-CSF induced splenic infarction has been reported in a patient diagnosed with triple-negative breast cancer and that was given a prophylactic filgrastim for 9 days. She was diagnosed as having a case of splenic infarction which was confirmed by radiological studies and biopsy and managed successfully with conservative approach.

In conclusion, splenic complications are considered rare but rather tragic and serious side effects of G-CSFs that should be addressed by physicians carefully. A close monitoring should be applied if this complication is suspected to curb further morbidity and mortality.
